# Age and gender differences in disabling foot pain using different definitions of the manchester foot pain and disability index

**DOI:** 10.1186/1471-2474-12-243

**Published:** 2011-10-26

**Authors:** Hylton B Menz, Tiffany K Gill, Anne W Taylor, Catherine L Hill

**Affiliations:** 1 Musculoskeletal Research Centre, Faculty of Health Sciences, La Trobe University, Bundoora, Victoria 3086, Australia; 2 Population Research and Outcome Studies, Department of Medicine, University of Adelaide, South Australia 5005, Australia; 3 Rheumatology Unit, Queen Elizabeth Hospital, Woodville, South Australia 5011, Australia; 4 The Health Observatory, University of Adelaide, Queen Elizabeth Hospital Campus, Woodville, South Australia 5011, Australia

## Abstract

**Background:**

The Manchester Foot Pain and Disability Index (MFPDI) has been used to determine the prevalence of disabling foot pain in several studies, however there is some debate as to which case definition is most appropriate. The objective of this study was to explore age and gender differences in the proportion of people with disabling foot pain using three different case definitions of the MFPDI and for each individual MFPDI item.

**Methods:**

A random sample of 223 participants aged 27 to 90 years (88 males and 135 females) from the North West Adelaide Health Study, who reported having pain, aching or stiffness in either of their feet on most days in the last month, completed the MFPDI by telephone interview. The proportion of people with disabling foot pain was determined using three definitions: (i) Definition A-at least one of the 17 items documented on at least some days in the last month; (ii) Definition B-at least one of the 17 items documented on most/every day(s) in the last month, and; (iii) Definition C-at least one of the ten functional limitation items documented on most/every day(s) in the last month. Cross-tabulations and chi-squared statistics were used to explore differences in responses to the MFPDI items according to age and gender.

**Results:**

The proportion of people with disabling foot pain according to each definition was as follows: Definition A (100%), Definition B (95.1%) and Definition C (77.6%). Definition C was most sensitive to age and gender differences. Exploration of individual MFPDI items indicated that age significantly affected both the pain intensity and functional limitation items, with younger people more likely to report their foot pain being worse in the morning, and older people more likely to report functional limitations. Although gender did not influence responses to the personal appearance items, women were more likely report functional limitations than men.

**Conclusions:**

Definition C of the MFPDI is more sensitive to age and gender differences in the proportion of people with disabling foot pain, and would therefore seem to be the most appropriate case definition to use in epidemiological studies involving a broad age range of participants.

## Background

Foot pain is common in older people, affecting 20 to 42% of those aged over 65 years [1-4]. The prevalence of foot pain in other age groups, however, has not been as widely studied, and a range of case definitions have been used. Wessex Feet, a population-based study of 700 people aged 0 to over 75 years in the UK, found that 41% reported foot 'problems', while the 1990 US National Health Interview Survey of 119,631 people aged over 18 years found that 24% of the sample reported foot 'trouble' [5]. More recently, we found that 17.4% of 3,206 people aged over 18 years who participated in the North West Adelaide Health Study (NWAHS) in Australia reported having 'pain, aching or stiffness' in either of their feet on most days in the last month [6].

One of the main limitations of previous epidemiological data on foot problem prevalence has been the absence of a validated assessment tool. However, in 2000, Garrow et al [7] developed the Manchester Foot Pain and Disability Index (MFPDI), which consists of 19 statements prefaced by the phrase 'Because of pain in my feet', formalised under four constructs: functional limitation (10 items), pain intensity (five items) and personal appearance (two items) and difficulties with work or leisure activities (two items). Each item is documented as being present 'none of the time', 'on some days' or 'on most/every day(s)'. Using the MFPDI, people reporting at least one item to be present 'on some days' are defined as having disabling foot pain [8]. The MFPDI has since undergone several psychometric evaluations [4,9-11] and has been used to determine the prevalence of disabling foot pain in three population-based studies [4,8,12].

A key advantage of the MFPDI is that it purports to measure *disabling *foot pain, and may theoretically be used to identify a more severely affected subgroup of people with foot pain. From an epidemiological perspective, it is important that these two subgroups (i.e. non-disabling foot pain *versus *disabling foot pain) can be identified, as they are likely to have different risk factor profiles and very different foot health care needs. However, Roddy et al [10] recently argued that the case definition originally proposed by Garrow et al [8] may not be appropriate, as it will include people with relatively mild foot problems and may therefore provide little additional discrimination beyond simply asking whether or not someone has foot pain. To address this, Roddy et al [10] proposed a revised definition in which disabling foot pain is considered to be present if one or more of the 10 functional limitation items are reported 'on most/every day(s)'. Applying this definition to a sample of 1,342 people aged over 50 years who reported foot pain in the last 12 months resulted in a prevalence of disabling foot pain of 74%, compared to 98% using the original definition.

The revised case definition proposed by Roddy et al [10] appears to be the most appropriate application of the MFPDI for prevalence studies. However, it remains unclear as to how the case definition performs when applied to a broader age range, as the sample used in the Roddy et al [10] study was limited to those aged over 50 years. Given that foot pain has a significant impact on functional ability in older people [1,13], it is likely that the prevalence of disabling foot pain using this definition (derived solely from the functional limitation items) will be lower in younger people. Furthermore, it is also likely that gender may influence responses to the MFPDI, however this is yet to be evaluated. Therefore, in order to determine whether the Roddy et al [10] definition of disabling foot pain is appropriate for use in a population-based sample of men and women aged 18 years and over, we conducted a preliminary study to explore the effect of age and gender on case definitions and individual item responses using the MFPDI.

## Methods

### Setting and study population

The North West Adelaide Health Study (NWAHS) was established in 2000 in the North-West region of Adelaide, South Australia [14]. The north-west region of Adelaide comprises approximately half of the population of the city of Adelaide and a third of the population of the state of South Australia. The regions also reflect the demographic profile of the state, covering a broad range of ages and socioeconomic areas. The study was designed in response to a need to assess the prevalence of priority conditions and examine their progression over time in a population-based community-dwelling cohort, to inform policy decisions about health care provision in South Australia.

Between June 2008 and August 2010, Stage 3 of the NWAHS was conducted. As part of the self administered questionnaire, respondents were asked "Over the past month, have you had pain, aching or stiffness in either of your feet on most days?" Participants who responded in the affirmative were considered to have foot pain. Respondents with foot pain were identified and a random sample of n = 387 undertook a telephone interview in January 2010. Given the delay between the Stage 3 survey and the telephone interview, participants were initially asked if they still had foot pain, Those who reported still having foot pain were then administered the MFPDI.

### Definitions of the MFPDI

The proportion of people with disabling foot pain was determined using three definitions of the MFPDI: (i) Definition A, the original case definition proposed by Garrow et al [8], which required at least one of the 17 items to be present on at least 'some days' in the last month; (ii) Definition B, which required at least one of the 17 items to be present on 'most/every day(s)' in the last month, and; (iii) Definition C, the definition proposed by Roddy et al [10], which required that at least one of the 10 functional limitation items to be documented on 'most/every day(s)' in the last month. Responses to each individual item of the MFPDI were also documented, with the exception of the two items relating to difficulties with work or leisure activities, as a large proportion of the sample were of retirement age.

### Statistical analysis

All analyses were undertaken using SPSS Version 15 and STATA Version 11.1. Frequencies were used to determine the proportion of people with disabling foot pain using the three definitions of the MFPDI and to explore responses to each individual MFPDI item. Data were cross-tabulated by age-group (27 to 50 years, 51 to 60 years, 61 to 70 years, and 71 years and over) and gender, and chi-squared (χ^2^) or Fisher's exact tests (where there were less than 5 cell counts) were applied. A significance level of p < 0.05 was used for all tests.

### Ethical approval

Ethical approval for the study was obtained from the Human Research Ethics Committee of the Queen Elizabeth Hospital, South Australia.

## Results

### Sample characteristics

**Figure 1:**
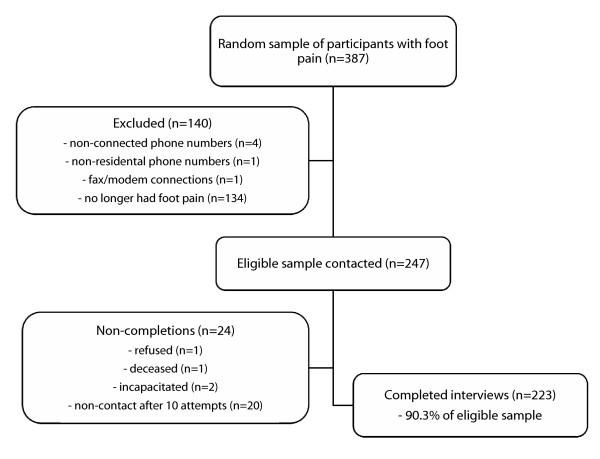
**Flow-chart of participant recruitment and response rate**.

A flow-chart of the sample recruitment and response rate is shown in Figure 1. Of the 387 randomly selected participants, 247 were deemed eligible, and of these, 223 completed the telephone interview (a response rate of 90.3%). The sample consisted of 88 males and 135 females aged 27 to 90 years (mean age 61.3 years, standard deviation 13.1).

### Proportion of people with disabling foot pain according to age and gender

**Figure 2:**
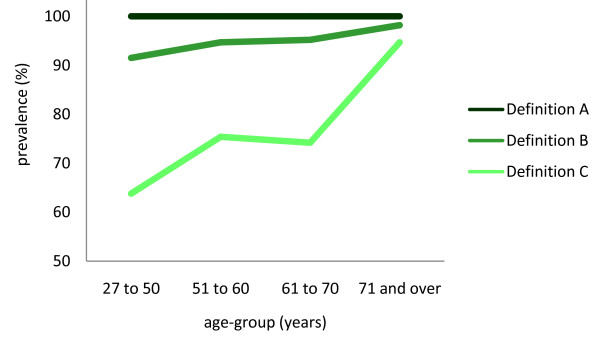
**Proportion of people with disabling foot pain according to age using three definitions of the MFPDI**.

The proportion of people with disabling foot pain using each definition was as follows: Definition A (100%), Definition B (95.1%) and Definition C (77.6%). Table 1 and Figure 2 show the prevalence of disabling foot pain according to age. Age was significantly associated with disabling foot pain prevalence using Definition C (Fisher's exact test, p = 0.001).

**Table 1:** Age differences in MFPDI foot pain definitions-n (%) [95% CI].

MFPDI definition	27 to 50 years (n = 47)	51 to 60 years (n = 57)	61 to 70 years (n = 62)	71 years and over (n = 57)	Significance
Definition A					
No disabling foot pain	0 (0)	0 (0)	0 (0)	0 (0)	-
Disabling foot pain	47 (100)	57 (100)	62 (100)	57 (100)	
Definition B					
No disabling foot pain	4 (8.5) [0.4-16.6]	3 (5.3) [-0.6-11.1]	3 (4.8) [-0.6-10.3]	1 (1.8) [-1.7-5.2]	p = 0.453†
Disabling foot pain	43 (91.5) [83.4-99.6]	54 (94.7) [88.9-100.6]	59 (95.2) [89.7-100.6]	56 (98.2) [94.8-101.7]	
Definition C					
No disabling foot pain	17 (36.2) [22.2-50.1]	14 (24.6) [13.2-35.9]	16 (25.8) [14.8-36.8]	3 (5.3) [0.06-11.1]	p = 0.001*†
Disabling foot pain	30 (63.8) [49.9-77.8]	43 (75.4) [64.1-86.8]	46 (74.2) [63.2-85.2]	54 (94.7) [88.9-100.6]	

* p < 0.05

†some cell counts n < 5, therefore Fisher's exact test applied

**Figure 3:**
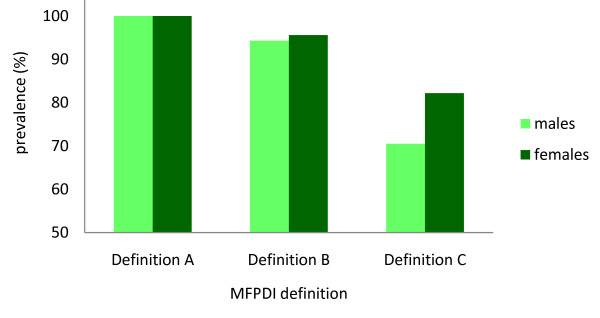
**Proportion of people with disabling foot pain according to gender using three definitions of the MFPDI**.

Table 2 and Figure 3 show the proportion of people with disabling foot pain according to gender. Female gender was significantly associated with the disabling foot pain using Definition C (χ^2 ^= 4.42, p = 0.039).

**Table 2:** Gender differences in MFPDI foot pain definitions-n (%) [95% CI].

MFPDI definition	Males (n = 88)	Females (n = 135)	Significance
Definition A			
No disabling foot pain	0 (0)	0 (0)	-
Disabling foot pain	88 (100)	135 (100)	
Definition B			
No disabling foot pain	5 (5.7) [0.8-10.6]	6 (4.4) [0.9-8.0]	p = 0.677
Disabling foot pain	83 (94.3) [89.4-99.2]	129 (95.6) [92.0-99.1]	
Definition C			
No disabling foot pain	26 (29.5) [19.9-39.2]	24 (17.8) [11.3-24.3]	p = 0.039*
Disabling foot pain	62 (70.5) [60.8-80.1]	111 (82.2) [75.7-88.7]	

* p < 0.05

### Responses to individual MFPDI items according to age and gender

Responses to the pain intensity, functional limitation and concern about personal appearance items of the MFPDI according to age are shown in Tables 3, 4 and 5. Age was significantly associated with one pain intensity item ('My feet are worse in the morning'), with participants aged 27 to 50 years more likely to report this being present on 'most/every day(s)' than older age-groups. Age was also significantly associated with several functional limitation items ('I walk slowly', 'I have to stop and rest my feet', ' I avoid hard or rough surfaces where possible' and 'I avoid standing for a long time'), with those aged 71 years and over more likely to report these being present on 'most/every day(s)'.

**Table 3:** Age differences in MFPDI pain intensity items-n (%).

MFPDI item	27 to 50 years (n = 47)	51 to 60 years (n = 57)	61 to 70 years (n = 62)	71 years and over (n = 57)	Significance
I still do everything but with more pain or discomfort					
None of the time	5 (10.6)	6 (10.5)	8 (12.9)	7 (12.5)	p = 0.834†‡
On some days	15 (31.9)	17 (29.8)	19 (30.6)	13 (23.2)	
On most/every days	27 (57.4)	34 (59.6)	35 (56.5)	36 (64.3)	
I have constant pain in feet					
None of the time	15 (31.9)	13 (22.8)	18 (29.0)	16 (28.1)	p = 0.576‡
On some days	16 (34.0)	17 (29.8)	19 (30.6)	15 (26.3)	
On most/every days	16 (34.0)	27 (47.4)	25 (40.3)	26 (45.6)	
My feet are worse in the morning					
None of the time	13 (27.7)	25 (43.9)	37 (61.7)	37 (66.1)	p = 0.001*†¥
On some days	13 (27.7)	12 (21.1)	10 (16.7)	4 (7.1)	
On most/every days	21 (44.7)	20 (35.1)	13 (21.7)	15 (26.8)	
My feet are more painful in the evening†					
None of the time	15 (31.9)	13 (22.8)	17 (27.9)	21 (37.5)	p = 0.553‡
On some days	13 (27.7)	18 (31.6)	14 (23.0)	14 (25.0)	
On most/every days	19 (40.4)	26 (45.6)	30 (49.2)	21 (37.5)	
I get shooting pains in my feet					
None of the time	20 (42.6)	23 (40.4)	29 (46.8)	30 (52.6)	p = 0.339‡
On some days	20 (42.6)	24 (42.1)	23 (37.1)	19 (33.3)	
On most/every days	7 (14.9)	10 (17.5)	10 (16.1)	8 (14.0)	

* p < 0.05

† "Don't know" responses excluded from analysis

‡χ^2 ^test (linear by linear)

¥ Some cell counts n < 5, so Fisher's exact test applied

**Table 4:** Age differences in MFPDI functional limitation items-n (%).

MFPDI item	27 to 50 years (n = 47)	51 to 60 years (n = 57)	61 to 70 years (n = 62)	71 years and over (n = 57)	Significance
I avoid walking outside at all					
None of the time	38 (80.9)	40 (70.2)	45 (72.6)	43 (75.4)	p = 0.163¥
On some days	9 (19.1)	14 (24.6)	10 (16.1)	8 (14.0)	
On most/every days	0 (0)	3 (5.3)	7 (11.3)	6 (10.5)	
I avoid walking long distances					
None of the time	22 (46.8)	13 (22.8)	25 (40.3)	16 (28.1)	p = 0.021*‡
On some days	16 (34.0)	18 (31.6)	14 (22.6)	11 (19.3)	
On most/every days	9 (19.1)	26 (45.6)	23 (37.1)	30 (52.6)	
I don't walk in a normal way					
None of the time	23 (48.9)	21 (37.5)	29 (46.8)	33 (57.9)	p = 0.603†‡
On some days	16 (34.0)	16 (28.6)	19 (30.6)	9 (15.8)	
On most/every days	8 (17.0)	19 (33.9)	14 (22.6)	15 (26.3)	
I walk slowly					
None of the time	21 (44.7)	23 (40.4)	22 (36.1)	16 (28.6)	p = 0.003*†‡
On some days	18 (38.3)	13 (22.8)	19 (31.1)	10 (17.9)	
On most/every days	8 (17.0)	21 (36.8)	20 (32.8)	30 (53.6)	
I have to stop and rest my feet					
None of the time	21 (44.7)	18 (31.6)	33 (53.2)	30 (52.6)	p < 0.001*¥
On some days	25 (53.2)	22 (38.6)	16 (25.8)	7 (12.3)	
On most/every days	1 (2.1)	17 (29.8)	13 (21.0)	20 (35.1)	
I avoid hard or rough surfaces where possible					
None of the time	21 (45.7)	24 (42.1)	28 (45.2)	16 (28.1)	p = 0.003*†‡
On some days	15 (32.6)	9 (15.8)	11 (17.7)	5 (8.8)	
On most/every days	10 (21.7)	24 (42.1)	23 (37.1)	36 (63.2)	
I avoid standing for a long time					
None of the time	14 (29.8)	9 (15.8)	19 (30.6)	12 (21.1)	p = 0.013*‡
On some days	20 (42.6)	21 (36.8)	12 (19.4)	7 (12.3)	
On most/every days	13 (27.7)	27 (47.4)	31 (50.0)	38 (66.7)	
I catch the bus or use the car more often					
None of the time	27 (58.7)	30 (52.6)	28 (45.2)	25 (43.9)	p = 0.636†‡
On some days	4 (8.7)	7 (12.3)	9 (14.5)	5 (8.8)	
On most/every days	15 (32.6)	20 (35.1)	25 (40.3)	27 (47.4)	
I need help with housework/shopping					
None of the time	44 (93.6)	45 (78.9)	54 (87.1)	43 (75.4)	p = 0.188†‡
On some days	1 (2.1)	8 (14.0)	5 (8.1)	9 (15.8)	
On most/every days	2 (4.3)	4 (7.0)	3 (4.8)	5 (8.8)	
I get irritable when my feet hurt					
None of the time	19 (40.4)	15 (26.3)	28 (45.9)	27 (47.4)	p = 0.319†‡
On some days	19 (40.4)	28 (49.1)	22 (36.1)	18 (31.6)	
On most/every days	9 (19.1)	14 (24.6)	11 (18.7)	12 (21.1)	

* p < 0.05

† "Don't know" responses excluded from analysis

‡ χ^2 ^test (linear by linear)

¥Some cell counts n < 5, so Fisher's exact test applied

**Table 5:** Age differences in MFPDI personal appearance items-n (%).

MFPDI item	27 to 50 years (n = 47)	51 to 60 years (n = 57)	61 to 70 years (n = 62)	71 years and over (n = 57)	Significance
I feel self-conscious about my feet					
None of the time	28 (59.6)	37 (64.9)	50 (80.6)	39 (68.4)	p = 0.178†‡
On some days	9 (19.1)	6 (10.5)	7 (11.3)	7 (12.3)	
On most/every days	10 (21.3)	14 (24.6)	5 (8.1)	11 (19.3)	
I get self-conscious about the shoes I have to wear					
None of the time	30 (63.8)	40 (70.2)	42 (67.7)	38 (66.7)	p = 0.487†‡
On some days	9 (19.1)	5 (8.8)	11 (17.7)	13 (22.8)	
On most/every days	8 (17.0)	12 (21.1)	9 (14.5)	6 (10.5)	

† "Don't know" responses excluded from analysis

‡ χ^2 ^test (linear by linear)

Responses to the pain intensity, functional limitation and concern about personal appearance items of the MFPDI according to gender are shown in Tables 6, 7 and 8. Gender was associated with the pain intensity item 'My feet are more painful in the evening', and three functional limitation items ('I don't walk in a normal way', 'I avoid hard or rough surfaces where possible' and 'I need help with housework/shopping'), with females more likely to report these being present on 'most/every day(s)'.

**Table 6:** Gender differences in MFPDI pain intensity items-n (%).

MFPDI item	Males (n = 88)	Females (n = 135)	Significance
I still do everything but with more pain or discomfort			
None of the time	12 (13.6)	14 (10.4)	p = 0.149†
On some days	19 (21.6)	45 (33.6)	
On most/every days	57 (64.8)	75 (56.0)	
I have constant pain in feet			
None of the time	24 (27.3)	38 (28.1)	p = 0.969
On some days	26 (29.4)	41 (30.4)	
On most/every days	38 (43.2)	56 (41.5)	
My feet are worse in the morning			
None of the time	45 (51.1)	67 (50.8)	p = 0.353†
On some days	12 (13.6)	27 (20.5)	
On most/every days	31 (35.2)	38 (28.8)	
My feet are more painful in the evening			
None of the time	32 (36.8)	34 (25.4)	p = 0.026*†
On some days	15 (17.2)	44 (32.8)	
On most/every days	40 (46.0)	56 (41.8)	
I get shooting pains in my feet			
None of the time	49 (55.7)	53 (39.3)	p = 0.055
On some days	28 (31.8)	58 (43.0)	
On most/every days	11 (12.5)	24 (17.8)	

* p < 0.05

† "Don't know" responses excluded from analysis

**Table 7:** Gender differences in MFPDI functional limitation items-n (%).

MFPDI item	Males (n = 88)	Females (n = 135)	Significance
I avoid walking outside at all			
None of the time	64 (72.7)	102 (75.6)	p = 0.883
On some days	17 (19.3)	24 (17.8)	
On most/every days	7 (8.0)	9 (6.7)	
I avoid walking long distances			
None of the time	34 (38.6)	42 (31.1)	p = 0.140
On some days	17 (19.3)	42 (31.1)	
On most/every days	37 (42.0)	51 (37.8)	
I don't walk in a normal way			
None of the time	51 (58.0)	55 (41.0)	p = 0.034*†
On some days	17 (19.3)	43 (32.1)	
On most/every days	20 (22.7)	36 (26.9)	
I walk slowly			
None of the time	35 (40.2)	47 (35.1)	p = 0.058†
On some days	16 (18.4)	44 (32.8)	
On most/every days	36 (41.4)	43 (32.1)	
I have to stop and rest my feet			
None of the time	44 (50.0)	58 (43.0)	p = 0.252
On some days	22 (25.0)	48 (25.6)	
On most/every days	22 (25.0)	29 (21.5)	
I avoid hard or rough surfaces where possible			
None of the time	46 (52.3)	43 (32.1)	p = 0.011*†
On some days	13 (14.8)	27 (20.1)	
On most/every days	29 (33.0)	64 (47.8)	
I avoid standing for a long time			
None of the time	28 (31.8)	26 (19.3)	p = 0.087
On some days	23 (26.1)	37 (27.4)	
On most/every days	37 (42.0)	72 (53.3)	
I catch the bus or use the car more often			
None of the time	50 (56.8)	60 (44.8)	p = 0.209†
On some days	8 (9.1)	17 (12.7)	
On most/every days	30 (34.1)	57 (42.5)	
I need help with housework/shopping			
None of the time	81 (92.0)	105 (77.8)	p = 0.018*
On some days	5 (5.7)	18 (13.3)	
On most/every days	2 (2.3)	12 (8.9)	
I get irritable when my feet hurt			
None of the time	42 (48.3)	47 (34.8)	p = 0.133†
On some days	30 (34.5)	57 (42.2)	
On most/every days	15 (17.2)	31 (23.0)	

* p < 0.05

† "Don't know" responses excluded from analysis

**Table 8:** Gender differences in MFPDI personal appearance items-n (%).

MFPDI item	Males (n = 88)	Females (n = 135)	Significance
I feel self-conscious about my feet			
None of the time	68 (77.3)	86 (63.7)	p = 0.096
On some days	9 (10.2)	20 (14.8)	
On most/every days	11 (12.5)	29 (21.5)	
I get self-conscious about the shoes I have to wear			
None of the time	67 (76.1)	83 (61.5)	p = 0.065
On some days	12 (13.6)	26 (19.3)	
On most/every days	9 (10.2)	26 (19.3)	

## Discussion

The objective of this study was to explore age and gender differences in of the proportion of people with disabling foot pain using three different case definitions of the MFPDI and for each of the individual MFPDI items, in order to determine the most appropriate use of this tool in a population-based sample of people aged 18 years and over. Applying the original case definition proposed by Garrow et al [8] (Definition A) to our sample of 223 participants who reported foot pain, aching, or stiffness in either of their feet on most days in the last month, the proportion of people with disabling foot pain was 100%. This finding is similar to Roddy et al [10], who found that this definition classified 98% of 1,342 people aged over 50 years who reported foot pain in the previous year as having disabling foot pain. We therefore concur with Roddy et al [10] that the original MFPDI case definition does not appear to distinguish between disabling and non-disabling foot pain, and provides essentially the same result as simply asking whether or not someone has foot pain. Importantly, this also appears to be true for the broader age range of our sample.

Applying Definition B, which requires at least one of the 17 items to be present on 'most/every day(s)' rather than on 'some days' in the last month resulted in a slightly lower proportion (95%), while applying Definition C, which requires at least one of the functional limitation items to be present on 'most/every day(s)', resulted in a substantially lower proportion (78%). These findings are also similar to Roddy et al [10], who found a prevalence of disabling foot pain of 74% when only the functional limitation items were considered, confirming the assertion that the inclusion of pain intensity and appearance constructs in a definition of disabling foot pain (as with Definitions A and B) may result in over-reporting [10]. Therefore, if the objective is to identify people with physical disability associated with foot pain, it would seem appropriate to use a case definition that focuses on functional impairment rather than pain intensity or concern about appearance.

A novel aspect of our study is that we explored the influence of age and gender on the proportion of people with disabling foot pain and the responses to individual MFPDI items. Given that it is likely that foot pain has a more pronounced impact on functional ability in older people compared to younger people, we expected that the number of participants classified as having disabling foot pain would increase across the four age-groups studied. As shown in Figure 2, the more stringent the definition of disabling foot pain, the greater the effect of age, which provides additional validation of Definition C for use in population-based studies with a broad age range. Analysis of the individual MFPDI items indicates that the higher prevalence of disabling foot pain using this definition is driven by an increased frequency of responses to the functional limitation items 'I walk slowly', 'I have to stop and rest my feet', 'I avoid hard or rough surfaces where possible' and 'I avoid standing for a long time' by older participants. This observation is consistent with the self-reported difficulties with walking and performing instrumental activities of daily living in older people with foot pain identified in previous studies [1,15,16].

As expected, the proportion of people with disabling foot pain was also influenced by gender, and our findings indicate that the greatest delineation between males and females was provided by Definition C (Figure 3). Interestingly, gender did not influence responses to the individual MFPDI pain intensity or personal appearance items, however females were more likely to report the functional limitation items 'I avoid hard or rough surfaces where possible' and 'I need help with housework/shopping' being present 'most/every day(s)' than males. Difficulty walking on hard or rough surfaces may be a more common problem for women with foot pain due to differences in footwear, with female footwear generally being more constrictive than male footwear [17]. The higher proportion of difficulties with housework or shopping, however, may merely reflect the fact that women perform more housework than men [18], and are therefore more likely to report that foot pain interferes with the performance of these tasks.

The findings of this study need to be considered in the context of several limitations. Firstly, this was an exploratory pilot study and was performed on a small subset of the overall NWAHS sample. As such, the findings may not necessarily be generalisable to the entire sample and we would therefore caution against interpreting these data as true prevalence estimates. Secondly, it is likely that there is an interaction between age and gender on prevalence of disabling foot pain, but due to the small sample we were unable to analyse this. Finally, we explored the MFPDI within a sample of people who had reported having pain, aching or stiffness in either of their feet on most days in the last month, and it is possible that different patterns would be evident if a different screening question was used.

## Conclusion

Different case definitions of the MFPDI result in different estimates of the proportion of people with disabling foot pain. Given that Definition C provides a more conservative estimate of disabling foot pain and is more sensitive to age and gender differences, it may be the most appropriate case definition to use in epidemiological studies involving a broad age range of participants.

## Competing interests

The authors declare that they have no competing interests.

## Authors' contributions

CLH, TKG, HBM and AWT conceived the study design, TKG conducted the statistical analysis, HBM and CLH interpreted the results and drafted the manuscript, and all authors read and approved the final manuscript.

## Pre-publication history

The pre-publication history for this paper can be accessed here:

http://www.biomedcentral.com/1471-2474/12/243/prepub
